# Total sleep deprivation reduces top-down regulation of emotion without altering bottom-up affective processing

**DOI:** 10.1371/journal.pone.0256983

**Published:** 2021-09-02

**Authors:** Anthony R. Stenson, Courtney A. Kurinec, John. M. Hinson, Paul Whitney, Hans P. A. Van Dongen

**Affiliations:** 1 Department of Psychology, Washington State University, Pullman, WA, United States of America; 2 Sleep and Performance Research Center, Washington State University, Spokane, WA, United States of America; 3 Elson S. Floyd College of Medicine, Washington State University, Spokane, WA, United States of America; Julius-Maximilians-Universität Würzburg, GERMANY

## Abstract

Sleep loss is reported to influence affective processing, causing changes in overall mood and altering emotion regulation. These aspects of affective processing are seldom investigated together, making it difficult to determine whether total sleep deprivation has a global effect on how affective stimuli and emotions are processed, or whether specific components of affective processing are affected selectively. Sixty healthy adults were recruited for an in-laboratory study and, after a monitored night of sleep and laboratory acclimation, randomly assigned to either a total sleep deprivation condition (*n* = 40) or a rested control condition (*n* = 20). Measurements of mood, vigilant attention to affective stimuli, affective working memory, affective categorization, and emotion regulation were taken for both groups. With one exception, measures of interest were administered twice: once at baseline and again 24 hours later, after the sleep deprived group had spent a night awake (working memory was assessed only after total sleep deprivation). Sleep deprived individuals experienced an overall reduction in positive affect with no significant change in negative affect. Despite the substantial decline in positive affect, there was no evidence that processing affectively valenced information was biased under total sleep deprivation. Sleep deprived subjects did not rate affective stimuli differently from rested subjects, nor did they show sleep deprivation-specific effects of affect type on vigilant attention, working memory, and categorization tasks. However, sleep deprived subjects showed less effective regulation of negative emotion. Overall, we found no evidence that total sleep deprivation biased the processing of affective stimuli in general. By contrast, total sleep deprivation appeared to reduce controlled processing required for emotion regulation.

## Introduction

Sleep loss is associated with a range of neurobiological and behavioral impairments that are commonly presented as reduced alertness, altered mood, and degraded task performance. The primary focus of laboratory studies of the effects of total sleep deprivation (TSD) has been on ‘cold’ (i.e., non-affective) cognitive tasks, including tasks involving vigilant attention, executive functions, and decision making [1–4]. Relatively little attention has been devoted to the effects of TSD on ‘hot’ (i.e., affective) processing. Most of the work that has focused on TSD and affect has examined mood, typically defined as a sustained affective state that is not elicited by specific stimuli and that varies in overall intensity. However, affect includes much more than mood. Affective processing encompasses states and processes that are characterized by differences in emotional valence (i.e., positive or negative feeling), arousal (i.e., high or low intensity), and motivational value (i.e., desirability based on seeking or avoiding), as well as psychological evaluations of these states [5,6]. In addition, affective processes include specific emotions, and momentary positive or negative affective reactions to stimuli.

In TSD research that has focused on mood, multiple studies have found a general decline in overall positive mood under total or partial TSD [6,7]. However, this finding may depend on how mood is assessed. In studies using the Positive and Negative Affect Schedule (PANAS) [8], which provides composite scores for overall positive and negative affect, TSD typically results in a decrease in reported positive affect rather than an increase in reported negative affect [9–11], while other measures [12] have found TSD to increase negative affect [13]. As time spent awake increases, subjects also tend to report increases in anger, anxiety, depression, fatigue, confusion, stress, and physical complaints, as well as decreases in vigor [6,14–16]. In part, these may be non-specific arousal effects associated with circadian rhythm and total time awake [17]. While general arousal level can affect cold cognitive tasks [18–20], effects of sleep loss on mood have been shown to be dissociable [e.g., 9].

Although the specific pattern of the relationship between TSD and mood is contingent on how mood is assessed, it is clear that sleep loss typically leads to changes in mood states [21]. The differences in mood during TSD have been presumed to be due to an underlying change in the processing of affective information, specifically a shift toward preferential processing of affectively negative stimuli relative to other types of stimuli [22–24]. This may be attributable to the effect of mood on processing of affective information. For instance, mood induction procedures outside of research on TSD have found that mood states can impact the bottom-up flow of affective information by biasing affective reactions to information [25–27], with these changes influencing the ease with which affective information is retrieved [28]. Mood states are also believed to prime affective evaluations both when individuals are evaluating internal states and in reference to stimuli in the environment [29,30]. Moreover, cold cognitive processes, such as attention and cognitive control, have been shown to be influenced by mood [31,32]. Thus, any changes in mood due to TSD may result in subsequent changes in processing of affective information. As a result, it is difficult to distinguish effects of sleep loss on affective processing from effects due to mood alone without statistically accounting for changes in mood. With some exceptions [33,34], controlling for changes in mood has not usually been done, casting doubt on the interpretability of extant literature.

Apart from the investigation of mood, researchers have also examined how TSD affects the processing of affective stimuli–i.e., those with emotional valence and intensity, but not associated with a specific emotional state. In these studies, which have primarily assessed the throughput of processing affective information, sleep deprived subjects have been found to display heightened physiological responses to emotional stimuli compared to their rested counterparts [9,35–37]. Additionally, reports on individuals under TSD have shown both preserved and impaired memory encoding for negatively valenced compared to neutral stimuli [38,39]. Sleep deprived individuals may also show deficits in emotion recognition and categorization [40–43]; for instance, sleep deprived subjects tend to incorrectly classify neutral stimuli as emotional [33,36,37]. However, in a few cases the ability to correctly recognize or categorize at least some emotions appeared to be preserved [44–46]. Together, these findings suggest that the bottom-up processing of affectively valenced stimuli, chiefly negative stimuli, may be heightened or maintained during TSD compared to more neutral stimuli, although the results vary depending on the stimuli and methodology employed.

The effects of TSD on affective processing during attention-demanding tasks, which involve the use of many non-affective processes, have received limited study. It has been reported that compared to well-rested controls, sleep deprived subjects are less accurate on working memory tasks that employ affectively valenced stimuli [34,47–50]. However, no published studies have directly investigated how TSD may influence attentional capture of affectively valenced stimuli. This gap in the literature is noteworthy because valenced stimuli generally capture attention more than neutral stimuli [51,52] and TSD has been consistently shown to impair vigilant attention [53,54]. In studies that indirectly assessed attention through event-related potentials (ERPs), sleep deprived individuals did show greater attention to emotional compared to neutral stimuli [46,55], but these studies assessed attention during emotion categorization tasks with limited attentional demands, rather than the sustained, goal-directed attention typically associated with vigilance.

Finally, in the pursuit of a goal, individuals may need to act on affective information rather than just experience it or attend to it. The study of goal-directed influences on affective processing occurs primarily through tasks assessing emotion regulation. Emotion regulation entails the modification of the perception and expression of affect in response to stimuli [56,57], often through the use of strategies that allow for voluntary moderation of the affective response [58–61]. Most of the work on TSD and emotion regulation has found that inadequate sleep reduces emotion regulation [62–64]. In a study where individuals were required to make either compatible or incompatible facial responses to emotional stimuli, subjects were able to regulate an affective response, but had slower response times overall [65]. Additionally, a recent meta-analysis reported small negative effects of sleep loss on emotion regulation in youth samples [21]. However, the use of self-report measures of sleep loss or emotion regulation in these studies may limit their interpretability [9].

It is possible that purported effects of TSD on emotion regulation reflect more general deficits in cold cognitive control processes [66,67]. General cognitive control is considered critical for effective emotion regulation [57,68], as many strategies for regulating emotions require the use of a goal-directed allocation of resources. For example, with the strategy of reappraising a situation, an elicited emotion is re-framed or re-contextualized in a less emotional context, and this process is typically seen as a more top-down and more effective emotion regulation strategy [69]. Imaging studies have shown that individuals engaging in reappraisal strategies produce greater activation in the prefrontal cortex than the amygdala, likely reflecting greater attentional control coinciding with a reduction of affective responding [70]. Even distracting oneself, another well-known strategy for emotion regulation, requires disengagement and shifting attention away from the emotional event, which also requires cognitive control processes. Thus, to accurately determine the source of impairment, TSD effects on cold cognitive control processes must be accounted for when evaluating TSD effects on emotion regulation.

To better understand what aspects of affective processing are, and are not, affected by TSD, we used a range of affective tasks to assess components of affective processing, and investigated whether TSD-associated changes in mood account for any observed changes in affective processing. Subjects were assigned to either a TSD condition or a rested control condition in a mixed between-within-subjects factorial design. We compared subjects’ rested and sleep deprived mood as well as their performance on tasks assessing three different aspects of affective stimulus processing (vigilant attention, working memory, and affective categorization) and a task assessing emotion regulation.

The novel purpose of this study was to answer three questions: (1) Is TSD biasing the processing of affective information when maintaining vigilant attention, updating working memory, or categorizing affective stimuli; (2) Is the ability to regulate emotion impaired under TSD; and (3) Are TSD effects on these different forms of affective processing dissociable from effects due to an overall change in mood? If TSD has a global biasing effect on affective processing, either as a result of or separately from TSD-induced mood changes, we should see valence-specific effects of TSD across our test battery. This global biasing of affective information should lead sleep deprived subjects to prioritize emotional, particularly negative, stimuli and rate non-valenced stimuli more emotionally. By contrast, if TSD affects mood without strongly biasing processing of affective stimuli generally, we should see typical TSD effects on cognition (e.g., lapses in vigilance) that would not interact with stimulus valence. In the case of emotion regulation, because it is inextricably bound with general cognitive control processes that are impacted by TSD, we would expect to see TSD effects regardless of whether TSD has general biasing effects on affective processing [71]. However, if emotion regulation impairments under TSD are entirely accounted for by changes in mood, this raises the possibility that changes in mood are the primary driver of typical TSD effects on cognitive control processes, as affect modulates these processes in non-sleep deprived samples [72].

## Material and methods

### Subjects

Sixty healthy adults (ages 22–37; 30 females) completed a laboratory study. They were screened extensively and found to be healthy and free of drugs, with normal or corrected to normal vision and hearing. Subjects were to refrain from caffeine, alcohol, and tobacco use in the week prior to and during the laboratory experiment. Additionally, subjects maintained their normal sleep schedule in the week leading up to the experiment, and adherence to sleep schedule was verified by sleep/wake diary, wrist-worn actigraphy, and called-in bedtimes and wake times. Polysomnography during the first night in the laboratory revealed no sleep-related abnormalities.

Subjects in this study were randomly assigned to a total sleep deprivation (TSD) condition or a well-rested (WR) control condition in a 2:1 ratio. The larger proportion of subjects in the TSD condition was needed to examine individual differences in attentional control during sleep deprivation separate from the present investigation. There were 40 subjects in the TSD and 20 in the WR condition. One subject’s data from the TSD condition were removed from analysis due to non-compliance (failure to follow instructions on several tasks and consuming caffeine during the week prior to the laboratory experiment), leaving a total of 59 subjects across the two conditions (39 in the TSD condition and 20 in the WR condition).

The study procedures were approved by the Institutional Review Board of Washington State University. All subjects provided written informed consent before beginning the experimental protocol and could withdraw from the study at any time.

### Procedure

**Fig 1:**
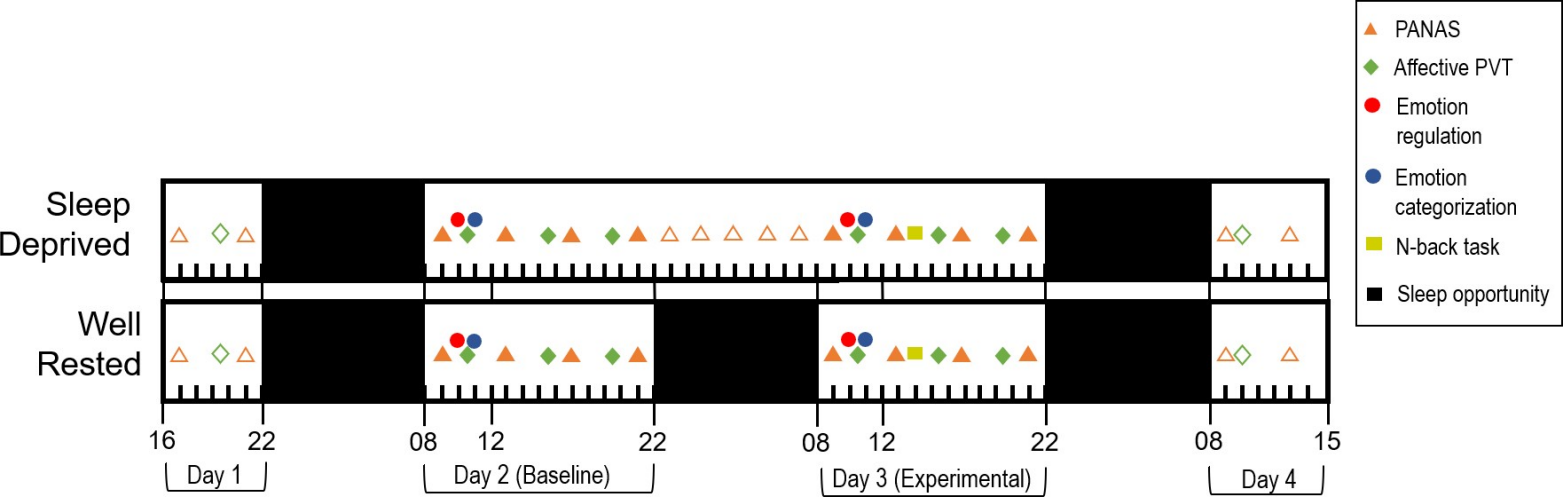
Study design for the total sleep deprivation condition (top) and well-rested control condition (bottom). Open symbols represent task administrations not used in analysis.

The study was conducted in the controlled laboratory setting of the Sleep and Performance Research Center at Washington State University, with fixed ambient temperature (22 ± 1°C) and fixed light levels during scheduled wake periods (< 100 lux). Subjects were in the laboratory for four consecutive days (three nights) (Fig 1). Upon arrival, they were randomly assigned to either the TSD condition or the WR condition (2:1 ratio). Subjects were not informed of their assignments until the evening of day 2. They were assigned to individual rooms for scheduled sleep periods and performance testing. Meals were provided every 4 h during scheduled wakefulness. Between sleep periods, meals, and performance testing, subjects could watch DVDs, play board or card games, and interact with other subjects and the research staff, but not engage in any vigorous physical or mental activity. Subjects did not have access to phones, computers, television, radio, or other means of interacting outside of the laboratory environment, and no visitors were allowed. Trained research assistants monitored subjects’ behavior throughout the study.

After arrival on day 1, subjects completed a practice test bout at 17:00, in which they completed practice trials for a standard test battery, including a standard 10-min psychomotor vigilance test (PVT) [52], the Karolinska Sleepiness Scale (KSS) [73], and the PANAS. This standard battery was administered every 4 h throughout the study during scheduled wake (09:00, 13:00, 17:00, and 21:00). At approximately 19:30 (*M* = 19:36, *SD* = 7 min) on day 1, subjects completed the first instance of the affective PVT (described below). During the first night in the laboratory, all subjects were acclimated to the laboratory setting and given a 10-h sleep opportunity (22:00 to 08:00).

On day 2, subjects completed the emotion regulation task (described below), which was administered during a morning test battery at around 09:45 (*M* = 09:41, *SD* = 3 min). They also completed the affective categorization task (described below) at around 11:15 (*M* = 11:21, *SD* = 9 min), and the affective PVT at approximately 10:30 (*M* = 10:38, *SD* = 7 min), 15:50 (*M* = 15:52, *SD* = 3 min), and 20:00 (*M* = 20:01, *SD* = 14 min). After condition assignments were given on the evening of day 2, subjects in the WR condition had a 10-h sleep opportunity overnight (22:00 to 08:00), whereas those in TSD condition were kept awake, with a nighttime test battery that included the standard PVT and the PANAS administered at 2-h intervals through the night (not used for analysis).

On day 3, subjects again completed the emotion regulation task (*M* = 09:45, *SD* = 2 min), the affective categorization task (*M* = 11:20, *SD* = 5 min), and the affective PVT (10:37 ± 4 min, 15:50 ± 3 min, and 19:57 ± 13 min)–i.e., at the same times of day as on day 2 but with 24 h of additional sustained wakefulness in the TSD condition. Subjects also performed the affective N-back task (described below) at around 14:00 (completion time *M* = 14:19, *SD =* 4 min), after approximately 30 h of sustained wakefulness for the sleep deprived subjects (6 h for the well-rested controls). At the end of day 3, all subjects were given a 10-h sleep opportunity (22:00 to 08:00).

On day 4, subjects again completed several test batteries, including the emotion regulation task (*M* = 09:45, *SD* = 4 min), the affective PVT (*M* = 10:33, *SD* = 3 min), and the affective categorization task (*M* = 11:16, *SD* = 4 min). Subjects left the laboratory in the afternoon on day 4. Given our primary interest in comparing subjects’ baseline (day 2) and experimental (day 3, WR or TSD) performance, we focused our analyses on data collected on days 2 and 3. These days are subsequently referred to as sessions 1 and 2, respectively.

### Materials

The instruments used in the current study and their corresponding outcome measures are shown in Table 1.

**Table 1:** List of instruments used in the current study, their corresponding outcome measures, and the expected effect of TSD.

Instrument	Outcome Variable(s)	Expected Effect of TSD
Positive and negative affect schedule (PANAS) [8]	Composite score of positive affect items Composite score of negative affect items	Decreased positive affect score No change in negative affect score
Affective psychomotor vigilance test (PVT)	Total number of attentional lapses (RT >500 ms)	Dampened increase in lapses on negative and/or positively valenced stimuli
Affective N-back task [77]	Accuracy (identifying if current trial was/was not matching stimulus from 1 or 2 trials back) RT	Greater accuracy and faster response times on negatively valenced stimuli
Affective categorization task [44]	Valence rating (-2 to +2) RT of categorization	Stimuli rated more negatively, especially for neutral images Faster RT to affectively valenced stimuli
Emotion regulation task [79]	Negativity rating (0 to 5)	Increased negativity rating during *attend/negative* condition

#### Positive and Negative Affect Schedule (PANAS)

The PANAS is a 20-item, self-report questionnaire designed to assess an individual’s mood states at a given time [8]. Ten items assess positive affect (e.g., *excited* and *determined*), and ten items assess negative affect (e.g., *irritable* and *afraid*). Subjects respond to each item using a 5-point Likert-type scale to indicate the degree to which they are experiencing certain emotions or feelings (1 = very slightly or not all, 5 = extremely). The PANAS has shown good internal consistency and adequate test-retest reliability for assessing mood at the present moment [8,74]. Outcomes of interest are a composite score for both positive and negative affect.

#### Affective Psychomotor Vigilance Test (PVT)

The PVT is a 10-min long sustained attention task [75]. During the task subjects must respond, via button press, as quickly as possible to stimuli that appear on the screen at random intervals between 2 and 10 s. Typically, the stimuli used in this task are a millisecond counter or simple neutral objects. However, during this experiment the stimuli were drawn from 300 words of the Affective Norms for English Words (ANEW) list [76]. With word valence rated from 1 = most displeasurable to 9 = most pleasurable, the 300 words were evenly divided into positive (*M* = 7.70, *SD* = 0.40), neutral (*M* = 5.30, *SD* = 0.52), and negative (*M* = 2.39, *SD* = 0.44) valence categories. With arousal rated from 1 = calm to 9 = excited, the words ranged from 2.50 to 8.17, with significantly higher arousal ratings for the negative words (*M* = 5.73, *SD* = 1.01) and the positive words (*M* = 5.65, *SD* = 1.03) than the neutral words (*M* = 4.22, *SD* = 0.73), *p*s < 0.001. Subjects were shown a random subset of the words in each test bout. Equal numbers of words from the three valence categories were presented in every test bout, and the order of words from each valence category was randomized within the test bout. Words could potentially re-appear across affective PVT administrations over the course of the experiment, but no words were repeated within each test bout.

The outcome of interest in this task was the number of lapses, defined as response times longer than 500 ms, for each valence category. This was used to assess whether vigilant attention was biased toward the capture of affectively valenced stimuli. Performance at 10:30, 16:00, and 20:00 on day 2 (baseline) and at the same time points 24 h later on day 3 (experimental) was used for analysis. Due to technical issues, data from the final session of interest for this task are not available for 8 subjects (5 from the WR condition, 3 from the TSD condition).

#### Affective N-back task

A modified version of the N-back task was used. This version was designed to assess individuals’ ability to update and maintain affective information in the focus of attention [77]. Stimuli in this task were the 200 positively or negatively valenced words from the ANEW also used in the affective PVT (see above). During the task subjects were instructed to indicate whether the word on the current trial had the same affective valence as the word *n* trials back (1-back or 2-back). Subjects could not move on to the next trial until they made a response. During each test bout, subjects completed four blocks of 42 trials; blocks alternated between 1-back and 2-back, and block order was counterbalanced across subjects. Words used as stimuli were randomly selected and could re-appear across the four task blocks, but no words were repeated within a block.

Accuracy scores were derived from whether the subject correctly identified a match or a non-match on each trial. As there are no valid responses to the first trial during 1-back blocks or to the first two trials during 2-back blocks, these were removed before analysis, as were trials with a response time (RT) of less than 150 ms (1.21% of experimental trials). Accuracy scores and mean RT were used as outcome measures of subjects’ affective working memory ability.

#### Affective categorization task

In the affective categorization task from Cote and colleagues [46], subjects rated 165 images (60 negative, 60 positive, and 45 neutral) from the International Affective Picture Set (IAPS) [78]. For each image, subjects were instructed to rate the “emotional quality of the picture” as quickly and accurately as possible using a 5-point scale (2 = *very positive*, 1 = *slightly positive*, 0 = *neutral*, -1 = *slightly negative*, and -2 = *very negative*). The images were presented over three blocks, with 55 pictures per block (20 negative, 20 positive, and 15 neutral). The same images were used during each test bout, but the order was randomized. Images were displayed for 1,500 ms, with a random inter-trial interval of between 2 to 4 s.

Mean ratings were calculated for each valence category to determine the extent to which subjects correctly categorized emotional stimuli, and RTs were measured to assess how long it took subjects to rate the images. For analyses, we excluded trials with no response or responses that were less than 150 ms (2.65% of trials) and trials with RTs more than 2 SD greater than the mean (4.39% of trials). One subject from the TSD condition did not provide responses during session 2.

#### Emotion regulation task

The emotion regulation task was taken from McRae and colleagues [79]. In each trial, subjects received a prompt (displayed for 2 s) to either *attend* to or *decrease* their emotional response to an upcoming image. Images consisted of negatively and neutrally valenced items, taken from the IAPS [78], and were shown for 7 s immediately following the prompt. After being presented with the *attend* prompt, subjects were to respond naturally to the negative or neutral image by allowing themselves to experience whatever emotions the image elicited. After being presented with the *decrease/negative* prompt, subjects were instructed to think of something to tell themselves about the picture that would make them feel less negative about the image.

During a practice bout prior to the experimental test bout, subjects were briefly instructed on how to perform each trial type and trained to recontextualize negative images during the *decrease/negative* trials. This involved guiding the subjects through a series of trials where examples of recontextualizations of the images were given to the subjects as they observed the negative image. Unlike the original task [79], however, our implementation of the task did not explicitly instruct subjects on what emotion regulation strategy to use during the *decrease/negative* condition.

Negative images used in this task were chosen to be negatively valenced but not extreme in nature (valence rating *M* = 2.89, *SD* = 0.24, arousal rating M = 5.07, *SD* = 0.19). During each test bout, subjects rated 45 images, with 15 images each from one of three conditions: attend/neutral image, attend/negative image, and decrease/negative image. The same 45 images were used for each test bout, but order of presentation was randomized.

Immediately following each stimulus presentation, subjects rated how negatively they felt about the image on a 5-point Likert-type scale (1 = not at all, 5 = very much). To decrease the possibility that task demands influenced the ratings, subjects were instructed to give their honest assessment and reminded that, in the *decrease/negative* condition, they may fail to regulate the experienced affect. Subjects were reminded of this prior to each administration of the task. This is standard in contemporary use of emotion regulation tasks [79–81].

Mean ratings for the attend/negative and decrease/negative conditions were used to measure subjects’ changes in negative affect. At baseline (session 1) the attend/neutral ratings were used as a manipulation check against the attend/negative ratings to make sure negative stimuli were having the intended effect. We excluded trials with no response or responses that were less than 150 ms (2.01% of trials) and trials with RTs more than 2 SD greater than the mean (5.00% of trials).

### Statistical analyses

For the emotion regulation and affective categorization tasks, we ran linear mixed-effects ANOVA models with fixed effects for condition (TSD or WR), session (1 or 2), affective valence (negative, neutral, or positive), and their interactions. For the emotion regulation task, the main analysis was performed for the *attend*/*negative* and *decrease/negative* conditions separately in order to investigate effects of TSD on the bottom-up (*attend/negative*) and top-down (*decrease/negative*) processing of affect independently. For the affective N-back task, we ran linear mixed-effects ANOVA models with fixed effects for condition (TSD or WR), N-back type (1-back or 2-back), affective valence (negative or positive), and their interactions. For both the affective PVT and the PANAS, we ran linear mixed-effects ANOVA models with fixed effects for condition (TSD or WR), bout (6 time points for affective PVT, 8 time points for PANAS), affective valence (negative, neutral, or positive, for the affective PVT only), and their interactions. All models included a random effect over subjects for the intercept. The alpha level was set to 0.05. All fixed effects reported used the Satterthwaite approximation for degrees of freedom, and pairwise comparisons were two-tailed and Bonferroni adjusted as needed. Analyses were run in R version 4.0.2 using the lme4, afex, and emmeans packages [82–84].

As secondary analyses, we included PANAS positive and negative affect ratings as two separate time-varying covariates in the mixed-effects ANOVA models for the other instruments, to control for mood. The positive and negative affect ratings used in analyses were taken from the test bouts closest to the respective tasks’ administrations. For all tasks except the affective N-back and affective PVT, this was the 09:00 PANAS bout in sessions 1 and 2 (days 2 and 3); for the N-back this was the 13:00 PANAS bout in session 2 (day 3); for the affective PVT this was 9:00, 17:00, and 21:00 during sessions 1 and 2 (days 2 and 3). A likelihood ratio test was used to analyze the effect of the inclusion of the positive and negative affect covariates on TSD effects/interactions of interest.

## Results

### PANAS

**Fig 2:**
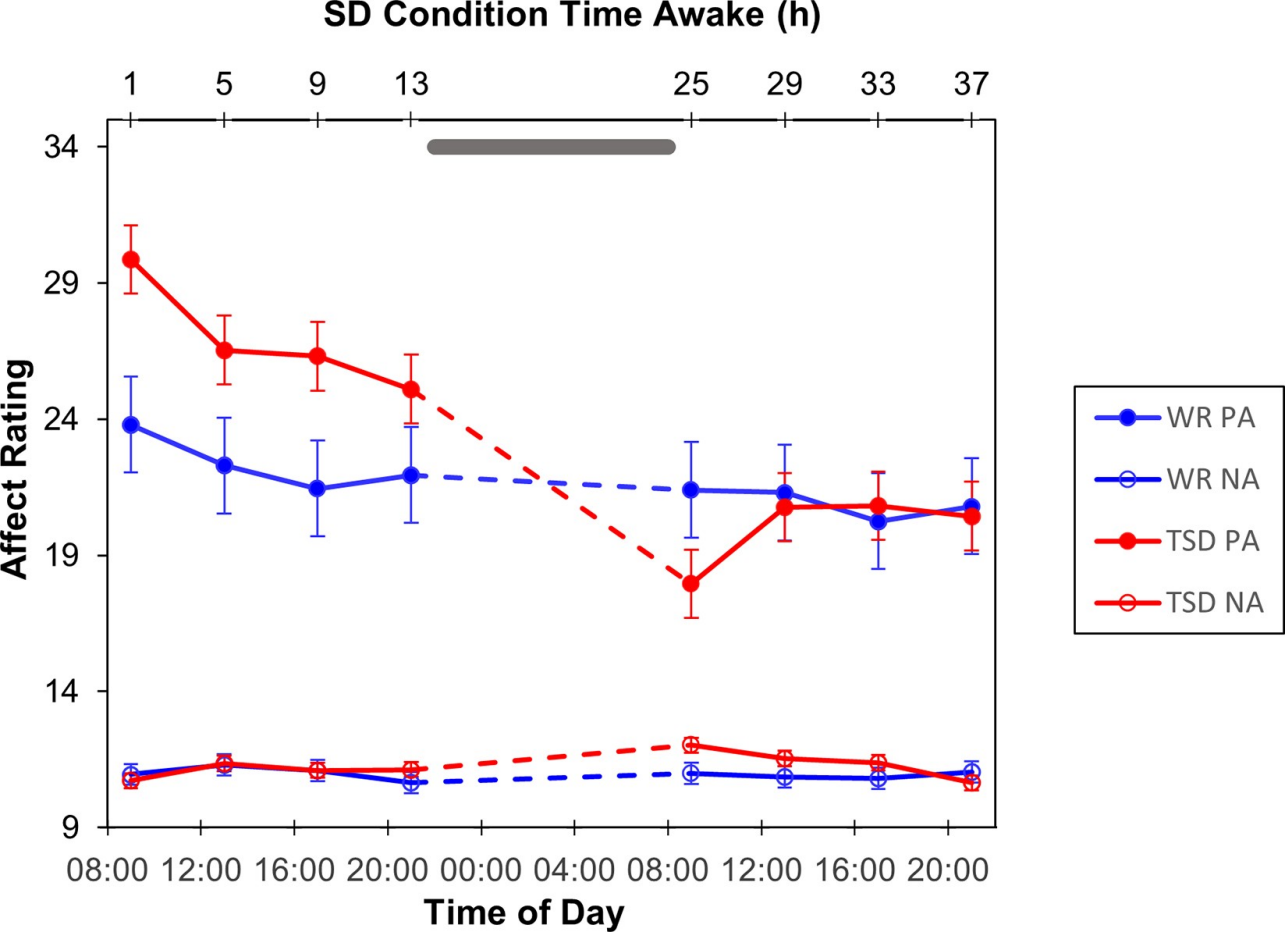
**Positive affect (PA) and negative affect (NA) ratings on the PANAS (mean ± SE) for subjects in the TSD (red) and WR (blue) conditions.** Grey bar represents sleep opportunity for WR condition and time awake for TSD condition.

For subjects’ self-ratings of affect on the PANAS, there was a significant interaction of condition by test bout for positive affect, *F*(7, 413) = 10.35, *p* < 0.001 (Fig 2). For sleep deprived subjects, positive affect scores decreased significantly over the course of the experiment, reaching the lowest point while subjects were sleep deprived. Planned contrasts confirmed post-deprivation scores were significantly lower than pre-deprivation scores within the sleep deprived group, all *p* < 0.001. The well-rested controls reported lower positive affect during the baseline day and experienced some loss of positive affect over the course of the experiment as well, but differences between test bouts were relatively small and not statistically significant for this group (for all contrasts, *p* > 0.08). There was also a significant interaction of condition by test bout for negative affect, *F*(7, 413) = 2.08, *p* = 0.044 (Fig 2). This interaction was driven by a difference during the first bout during the experimental day (25 h awake), where TSD subjects experienced a small but significant increase in negative affect, *t*(187) = 2.18, *p* = 0.030.

### Affective PVT

**Fig 3:**
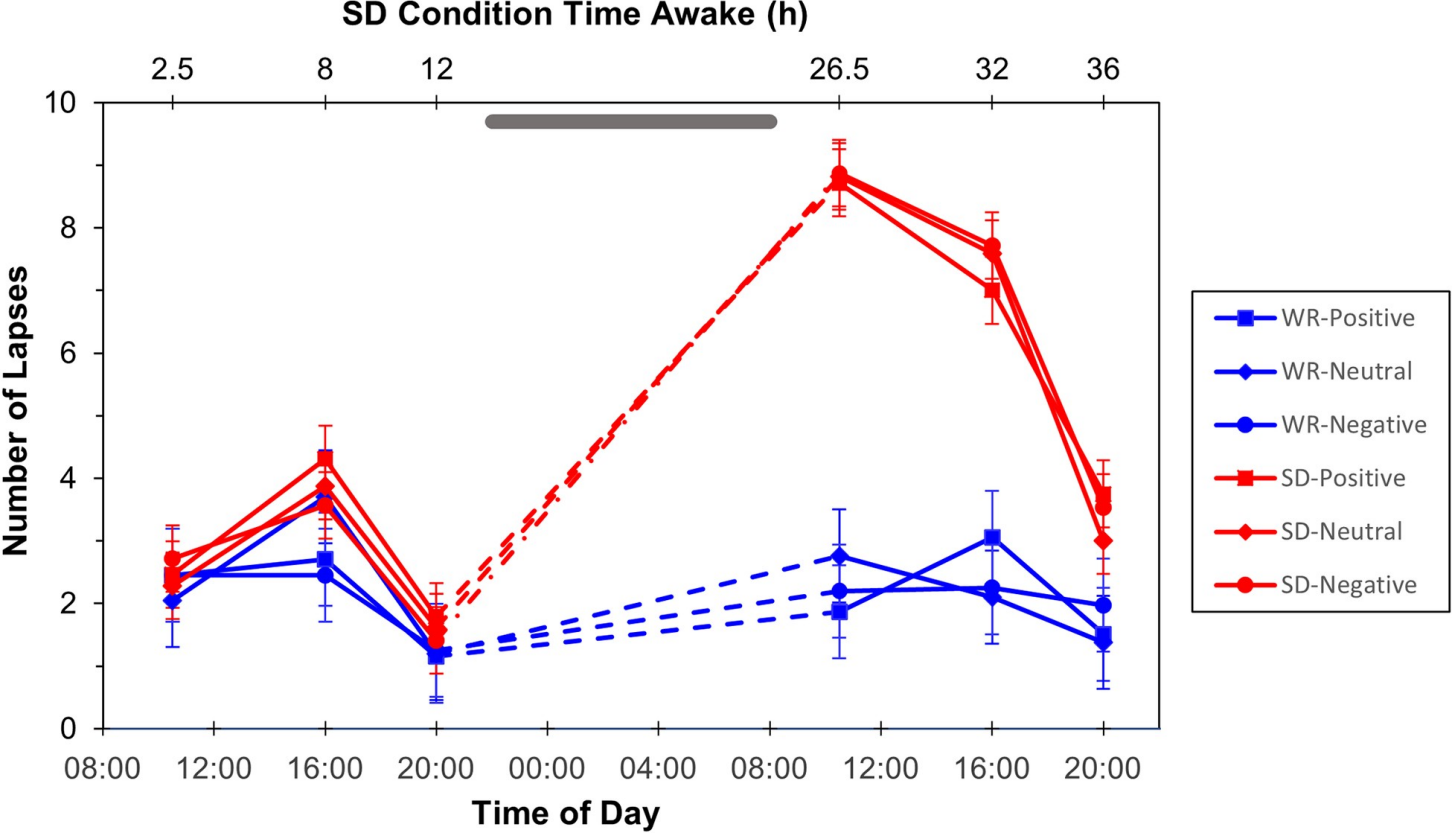
**Number of lapses (mean ± SE) on the affective PVT for each category of valenced word stimuli for the TSD (red) and WR (blue) conditions.** Grey bar represents sleep opportunity for WR condition and time awake for TSD condition.

For number of lapses on the affective PVT, there was an interaction of condition by test bout, *F*(5, 974.45) = 44.07, *p* < 0.001 (Fig 3). This effect was driven by differences between conditions test bouts during the experimental day, when the individuals in the TSD condition were sleep deprived and experienced significantly more lapses than the individuals in the WR condition, *t*(110) = 9.10, *p* < 0.001; *t*(105) = 7.01, *p* < 0.001; and *t*(123) = 2.44, *p* = 0.016, respectively. However, there were no significant main effects of stimulus valence on the number of lapses, *F*(2, 973.00) = 0.02, *p* = 0.983, nor was there a significant interaction of condition by test bout by valence, *F*(10, 973.00) = 0.69, *p* = 0.732, indicating that there were no differences in how the different valence types captured vigilant attention. Inclusion of positive and negative affect did not alter this pattern of results, nor were positive or negative affect significant as covariates, *F*(1, 713.88) = 1.39, *p* = 0.239, for positive affect; *F*(1, 1027.39) = 0.39, *p* = 0.535, for negative affect.

Since subjects in this task were not explicitly instructed to read the words presented as stimuli but simply to respond as quickly as possible, it is possible that subjects were not processing the words and therefore our affect manipulation could have been ineffective. However, a *post hoc* analysis found that although there was a strong correlation between RTs on the affective PVT and the standard PVT from the bout closest to the affective PVT, *r* = 0.70, *p* < 0.001, and RTs on the affective PVT were considerably slower than RTs on the standard PVT (Table 2). This slowing of RTs on the affective PVT likely indicates that subjects were processing the words. Additionally, including RTs on the standard PVT as a covariate in our primary analysis did not change the pattern of results despite RTs on the standard PVT being a significant covariate in the model, *F*(1, 924,59) = 261.08, *p* < 0.001.

**Table 2:** Means and standard deviations of RTs (ms) for the affective and standard PVT across study days.

Group	Study day	Affective PVT mean RT (± SD)	Standard PVT mean RT (± SD)
Well rested	1	367.12 (38.40)	286.79 (19.17)
	2	362.96 (39.13)	286.11 (23.11)
Total sleep deprivation	1	373.18 (42.98)	285.36 (27.01)
	2	479.68 (92.66)	348.58 (61.45)

### Affective N-back task

**Fig 4:**
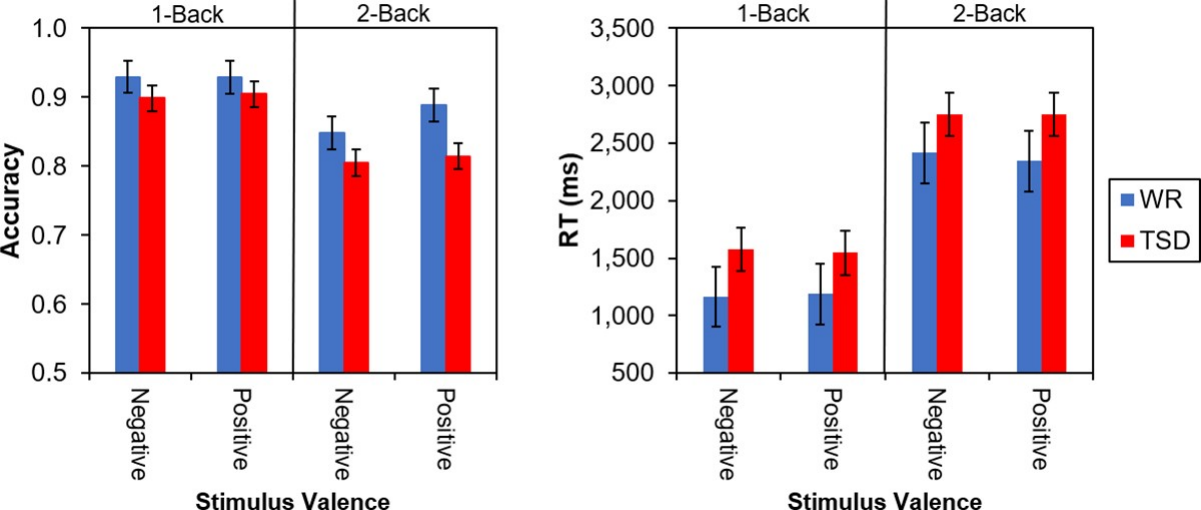
Accuracy (mean ± SE) on the 1- and 2-back blocks of the N-back task, for both positive and negative stimuli, in the TSD (red) and WR (blue) conditions (left). Response time for accurate trials (mean ± SE) on the 1- and 2-back blocks of the N-back task, for both positive and negative stimuli, in the TSD and WR conditions (right).

For accuracy scores on the affective N-back task, there was a significant main effect of N-back type, *F*(1, 177) = 65.26, *p* < 0.001. As expected, subjects were more accurate on 1-back than 2-back blocks, *t*(177) = 8.08, *p* < 0.001. The main effects of valence and condition were not significant, *F*(1, 177) = 2.17, *p* = 0.142, and *F*(1, 59) = 2.53, *p* = 0.117, respectively; and there were no significant interactions, including the condition by N-back type interaction, *F*(1, 177) = 2.74, *p* = 0.100, and the crucial condition by valence interaction, *F*(1, 177) = 0.41, *p* = 0.522 (Fig 4, left). RT analyses showed a similar pattern of results, with only N-back type having an effect, *F*(1, 177) = 200.02, *p* < 0.001, and no significant condition by valence interaction, *F*(1, 177) = 0.00, *p* = 0.963. Subjects were quicker to respond to 1-back trials than 2-back trials, *t*(177) = -14.14, *p* < 0.001 (Fig 4, right). Controlling for subjects’ positive and negative affect did not change this pattern of results, and neither positive nor negative affect were significant as covariates for accuracy, *p*s > 0.799, or response time, *p*s > 0.09.

### Affective categorization task

**Fig 5:**
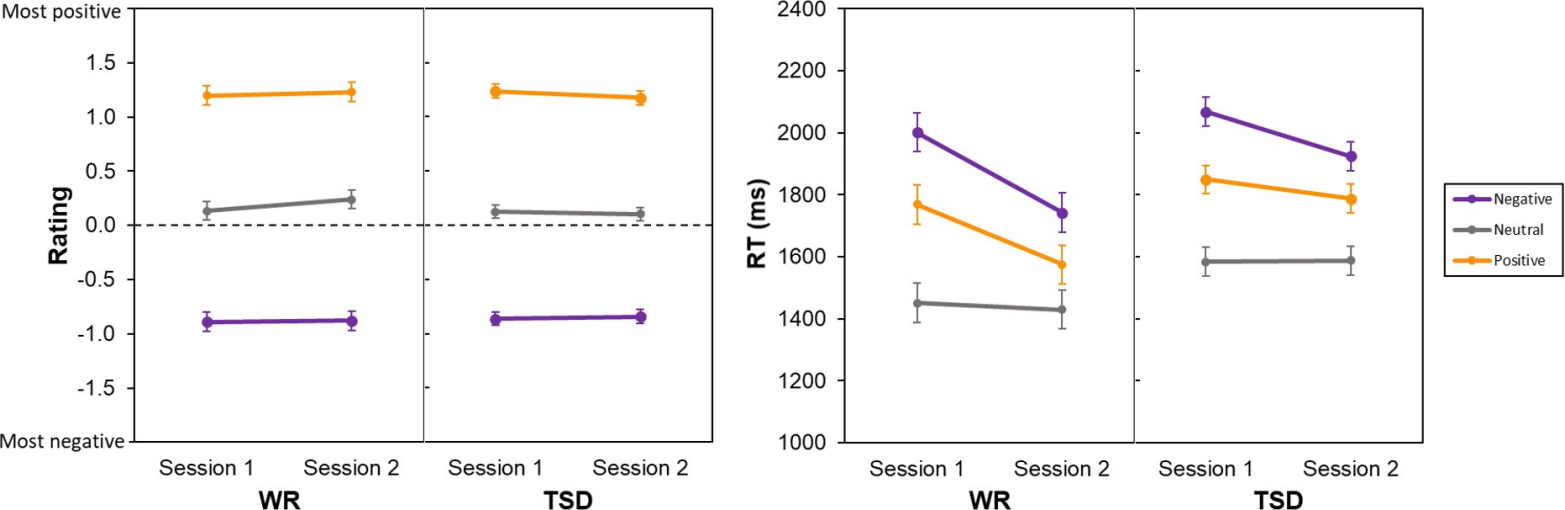
Valence ratings (mean ± SE) on the affective categorization task for each stimulus type, in the WR and TSD conditions (left). Response times (mean ± SE) on the affective categorization task for each stimulus type, in the WR and TSD conditions (right).

For the image ratings on the affective categorization task, there was a significant main effect of valence, *F*(2, 290) = 881.23, *p* < 0.001. As expected, positive images were rated significantly higher on the emotional quality scale than neutral images, *t*(290) = 21.39, *p* < 0.001, which in turn were rated significantly higher than negative ones, *t*(290) = 20.59, *p* < 0.001. There was no significant main effect for session, *F*(1, 290) = 0.11, *p* = 0.741, nor for condition, *F*(1, 58) = 0.07, *p* = 0.789, and there were no significant interactions, including the crucial condition by session by valence interaction, *F*(2, 290) = 0.26, *p* = 0.768 (Fig 5, left).

For RT, there were significant interactions of condition by session, *F*(1, 290) = 4.53, *p* = 0.034, and session by valence, *F*(2, 290) = 6.84, *p* = 0.001. There also were significant main effects of condition, *F*(1, 58) = 5.41, *p* = 0.024, session, *F*(1, 290) = 27.42, *p* < 0.001, and valence, *F*(2, 290) = 129.11, *p* < 0.001. Although all subjects across both groups responded faster in session 2 compared to baseline, *t*(290) = 5.24, *p* < 0.001, well-rested subjects were faster than sleep deprived subjects in session 2, *t*(73.87) = -2.91, *p* = 0.005. Separately, in both sessions, subjects responded more slowly to negative images than positive images, *t*(290) = 7.19, *p* < 0.001, and more slowly to positive images than neutral images, *t*(290) = 8.85, *p* < 0.001. However, subjects were faster at rating negative and positive images in session 2 than in session 1, *t*(290) = 5.41, *p* < 0.001, and *t*(290) = 3.44, *p* < 0.001, respectively. The crucial condition by session by valence interaction was not significant, *F*(2, 290) = 0.60, *p* = 0.8547 (Fig 5, right).

Including covariates for positive and negative affect did not alter the reported pattern of results for accuracy. For ratings on the emotion categorization task there was a significant effect of positive affect, *F*(1, 83.73) = 13.09, *p* < 0.001, but not negative affect, *F*(1, 166.63) = 1.13, *p* = 0.289. Including covariates for positive and negative affect also did not alter the reported pattern of results for RT, nor were positive and negative affect significant as covariates, *F*(1, 188.44) = 3.86, *p* = 0.051 and *F*(1, 338.23) = 1.35, *p* = 0.246, respectively.

### Emotion regulation task

As a manipulation check to ensure that subjects were attending to images and not simply responding arbitrarily, we first compared the *attend/neutral* condition and the *attend/negative* trial types. As expected, we found that negative stimuli were rated as significantly more negative than neutral stimuli, *F*(1,177) = 565.97, *p* < 0.001.

**Fig 6:**
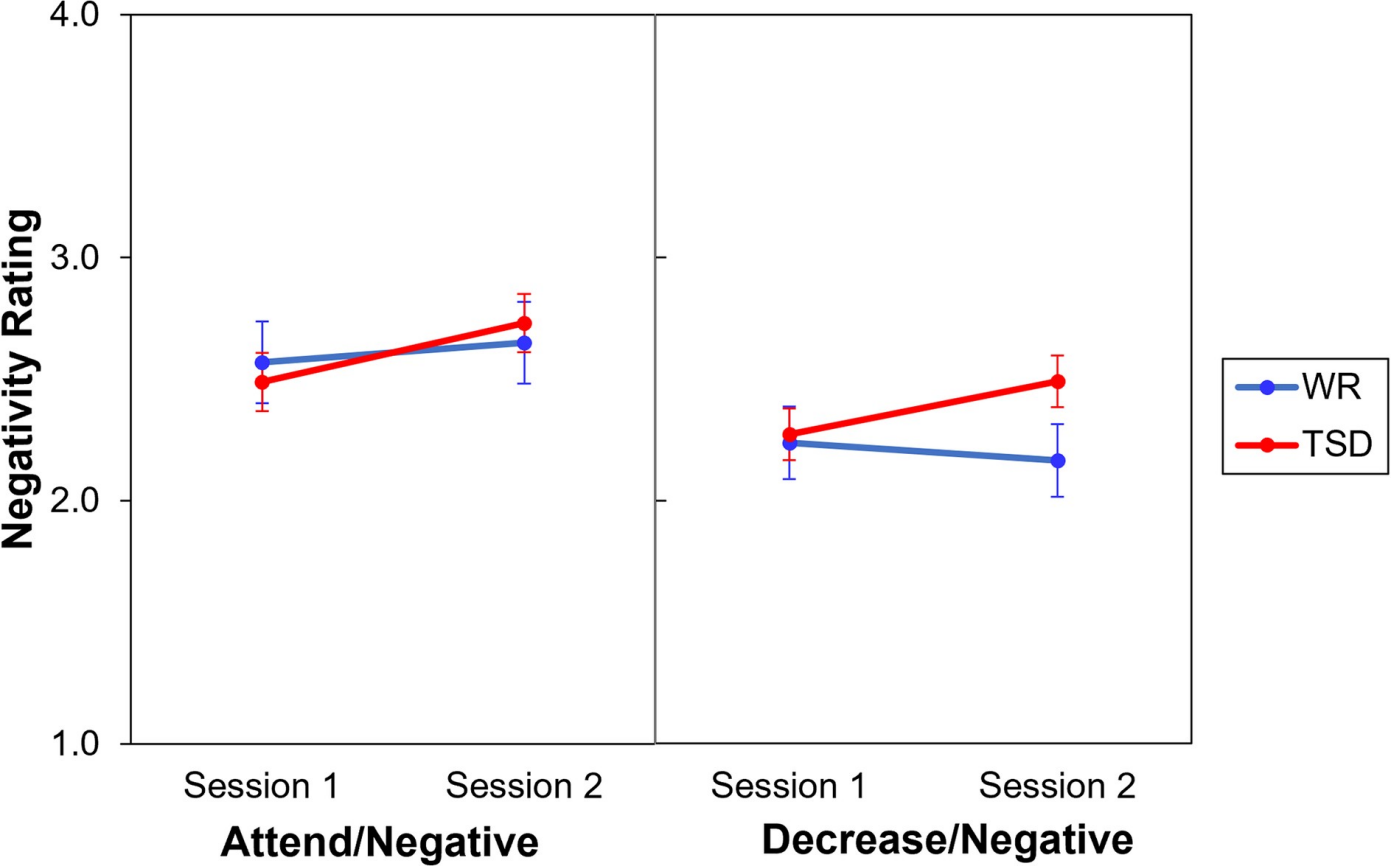
Negativity ratings (mean ± SE) during *attend/negative* trials (left) and *decrease/negative* trials (right) of the emotion regulation task, in the TSD (red) and WR (blue) conditions.

In order to determine whether subjects in the TSD condition experienced negative stimuli differently than subjects in the WR condition, we analyzed ratings for the *attend/negative* condition. There was a main effect of session on ratings of negative stimuli, with both well-rested controls and sleep deprived subjects rating images more negatively during session 2, *F*(1,59) = 7.33, *p* = 0.009. However, there was no effect of condition, *F*(1,59) = 0.00, *p* = 0.999, and no interaction of condition by session, *F*(1,59) = 1.84, *p* = 0.180, indicating that this change occurred regardless of study condition (Fig 6, left).

Next, to assess whether total sleep deprivation affected subjects’ ability to reduce the experience of negative affect, we analyzed ratings for the *decrease/negative* condition. There were no main effects of session, *F*(1,59) = 1.92, *p* = 0.171, or condition, *F*(1,59) = 1.11, *p* = 0.296. However, there was a significant interaction of condition by session, *F*(1, 59) = 7.55, *p* = 0.008 (Fig 6, right). When directed to decrease their emotions, sleep deprived individuals rated images as more negative during session 2 compared to session 1, *t*(59) = -3.55, *p* < 0.001.

After adding positive and negative affect as covariates in the model for the *decrease/negative* condition, the interaction of condition by session was no longer significant, *F*(1, 57.79) = 1.10, *p* = 0.298. The covariates trended to significance for both positive affect, *F*(1, 109.69) = 3.66, *p* = 0.058, and negative affect, *F*(1, 77.94) = 3.45, *p* = 0.067. Likelihood ratio tests revealed that the interaction between condition and session did not provide a significant improvement in goodness-of-fit compared to a model without the interaction, *χ*^*2*^(1) = 1.09, *p* = 0.221, whereas inclusion of the covariates did significantly improve goodness-of-fit, *χ*^*2*^(2) = 14.28, *p* < 0.001. Further, each covariate separately provided a significant improvement of goodness-of-fit compared to a model without each corresponding covariate, *χ*^*2*^(1) = 7.37, *p* = 0.004 for positive affect and *χ*^*2*^(1) = 6.81, *p* = 0.005 for negative affect.

**Fig 7:**
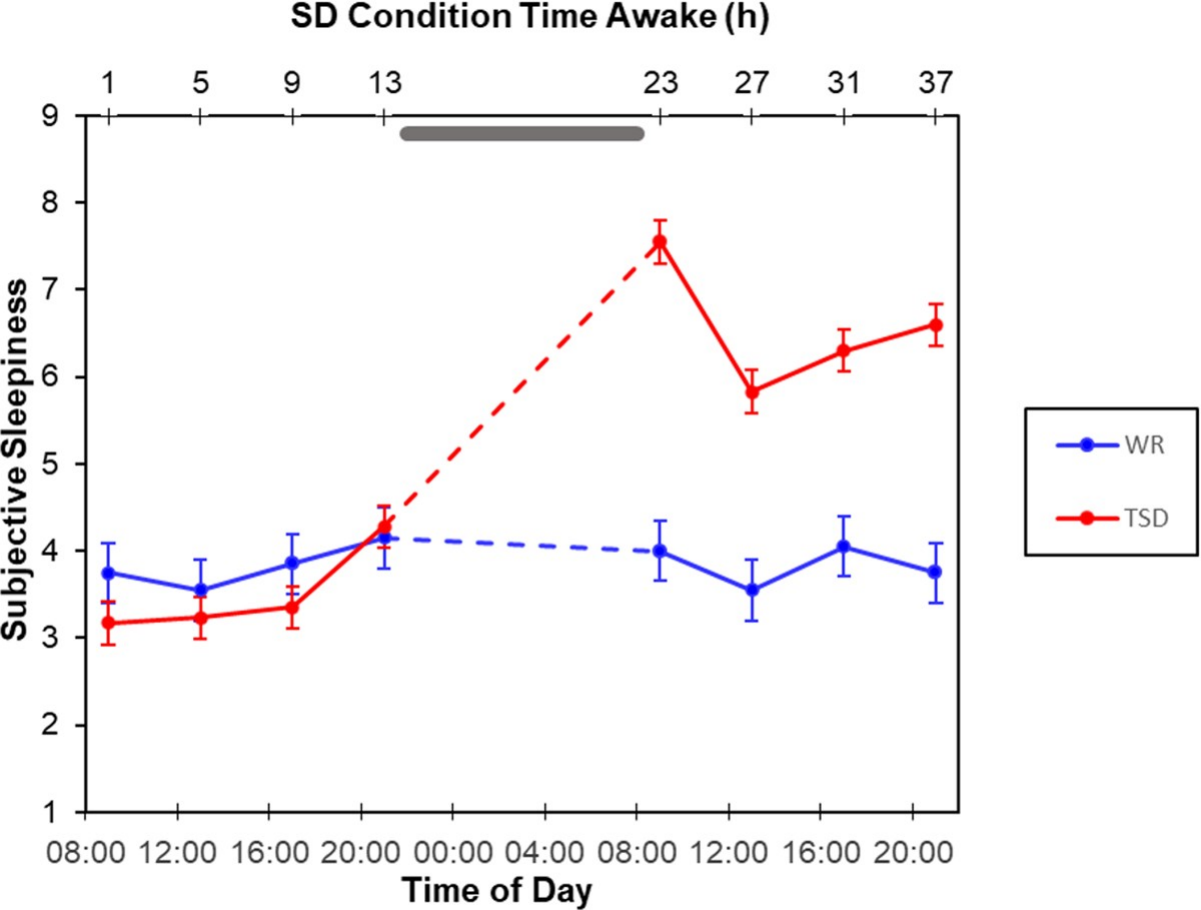
Subjective sleepiness ratings (mean ± SE) on the KSS for the TSD (red) and WR (blue) conditions. Grey bar represents sleep opportunity for WR condition and time awake for TSD condition.

To determine whether the PANAS covariates were tapping into changes in mood and not a more general factor related to subjective sleepiness, we ran the same analysis using scores on the KSS as a covariate. The KSS was chosen as it was administered at the same time as the PANAS, it does not ask about the subjects’ affective state, and, like the PANAS, is a self-report measure. Although self-reported sleepiness should be somewhat independent of self-reported mood, positive affect was moderately correlated with KSS ratings, *r* = -0.40, *p* < 0.001, while negative affect was weakly related to KSS ratings, *r* = 0.12, *p* = 0.009. Additionally, similar to the PANAS, a significant interaction of condition by test bout was observed for KSS scores, *F*(7, 420) = 23.64, *p* < 0.001, with the TSD group but not the WR group reporting increased subjective sleepiness during the experimental day (Fig 7).

Contrary to our expectations, including KSS scores as a covariate removed the once significant interaction, and KSS scores were a significant covariate, *F*(1, 87.83) = 8.69, *p* = 0.004. Additionally, likelihood ratio tests revealed that the observed interaction did not provide a significant improvement in goodness-of-fit above and beyond a model without the interaction, *χ*^*2*^(1) = 0.53, *p* = 0.419. That both self-reported affect and self-reported sleepiness had the same effect on our model weakens the argument that the effect of TSD on emotion regulation was primarily driven by TSD-induced changes in mood.

## Discussion

People who are sleep deprived show substantially impaired performance on tasks that require vigilance [54], and varying levels of impairment on other cognitive tasks [3]. They also show deficits on a variety of affective processing tasks [33,34,37,40,43], but the literature lacks clarity as to which components of affective processing are specifically affected by TSD, and whether the TSD effects on mood [9–11] account for any of the observed TSD effects on affective processing. We explored three questions related to TSD and affect: (1) whether TSD biases the processing of affective stimuli when maintaining vigilant attention, updating working memory, or categorizing affective stimuli; (2) whether emotion regulation is impaired by TSD; and (3) whether changes in mood account for TSD effects on affective processing.

To answer these questions, we assessed outcomes on multiple affective tasks, within a highly controlled environment, in a mixed within-between-subjects study design with a control group. Overall, we found that (1) sleep deprived individuals do not have impaired or otherwise biased processing of affective information, but (2) do show decrements in their ability to regulate negative emotional experience when directed to do so; and that (3) although sleep deprived subjects reported the expected decreases in positive affect as time awake increased, without any major increases in negative affect, we observed no significant effects of positive affect unique to the sleep deprived subjects apart from our results on the emotion regulation task. These findings indicate that the bottom-up flow of affective information appears to be relatively unaffected by TSD–however, top-down context-dependent regulation of affective information is degraded by TSD, in part due to a change in mood while sleep deprived.

We did not observe any interactions of TSD and stimulus valence on tasks that assessed the processing of affective information. In the affective PVT, sleep deprived subjects showed the expected increase in lapsing overall, but neither sleep deprived nor rested subjects showed any effect of stimulus valence on the number of lapses. If TSD were biasing the processing of affective information, we would have likely seen a difference between the number of lapses on valenced compared to neutral stimuli due to automatic attentional capture, but this was not the case. As vigilant attention is an integral process for many cognitive tasks, deficits in vigilance associated with TSD are likely to underlie impairments not specifically related to affective processing [54], as also seen on the working memory and affective categorization tasks.

Subjects under TSD did not demonstrate any noticeable impairments in their ability to update and maintain affective information once it entered the focus of attention. In line with previous work investigating emotional working memory [34,47–49], we did not find any effect of valence on subjects’ working memory performance. Had TSD biased affective processing towards preferential treatment of valenced information, differences in accuracy would have likely been present. Additionally, our sleep deprived subjects showed similar working memory performance as rested subjects, a finding consistent with work suggesting that differences in performance on working memory tasks between TSD and rested individuals are due to the non-executive components of the tasks rather than true working memory deficits [85,86].

With regard to recognizing and categorizing affective stimuli, much like Cote and colleagues [46] we found that sleep deprived subjects were slower to rate the emotional quality of the images overall, but this response slowing under TSD was not moderated by item valence. Additionally, we found that subjects undergoing TSD did not rate neutral images any differently than their well-rested counterparts. These results are in contrast to studies reporting that sleep deprived subjects rate neutral images as more emotional [33,36,37]. The discrepancy between our findings on sleep loss and affective categorization and those of others may be attributable to differences in methodology, as our findings are consistent with previous work using a similar method [35]. For instance, our subjects saw images rather than facial expressions, categorized the images into three rather than two valence categories, and made their ratings using a short, ordinal scale rather than a self-assessment manikin [87].

Although positive and negative PANAS scores were not always significant covariates and did not change the pattern of results for most of our tasks, mood did have a significant effect on the affective categorization task. Specifically, change in positive affect was a significant covariate for performance in ratings of affective stimuli. However, the pattern of results did not change. In a previous study reporting that TSD affected how subjects rated neutral images, subjects showed an increase in negative mood as measured using visual analog scales [26]. Negative mood was not a significant covariate in that study, but it does suggest that changes in mood may account for some variance in how subjects rate images, depending on how mood is assessed.

Taken as a whole, results from these three tasks indicate that the ability to attend to, maintain, and categorize affective information was not substantially influenced by one night of TSD. Contrary to what has been reported in previous work, we failed to observe a significant effect of sleep deprivation on how affective information was processed. With 39 subjects in the TSD condition and 20 in the WR condition, the sample size was more than adequate to demonstrate TSD effects and interactions for the various aspects of cognition that are known to be affected, such as emotion regulation, vigilant attention, and mood [6,7,21,54,62–64]. If the effects of sleep deprivation on bottom-up affective processing were of the same magnitude as the effects found in our other cognitive tasks, then we should have had no difficulty detecting these effects. Nor was there an insufficient amount of TSD; the duration of wakefulness for sleep deprived subjects in our study was comparable to that used in previous work where profound effects and interactions of TSD were observed [33,36,42]. Through the use of tasks that assess different components of the processing of affective information, this study provides evidence that the bottom-up flow of affective information remained unabated during sleep deprivation.

Results from the *attend/negative/neutral* trials of the emotion regulation task provide further support for the contention that one night of TSD does not impair bottom-up affective processing of affect. All subjects rated negative images as more negative when attending to these stimuli during the second session, regardless of study condition. Furthermore, during the experimental day, negativity ratings of both neutral and negative images that were attended to did not differ between the two groups, suggesting that these images were experienced similarly. In conjunction with the aforementioned results, we find that any changes experienced under TSD are unlikely to be the result of disruptions to the bottom-up processing of affective information.

Using a commonly accepted emotion regulation task, we obtained results that are consistent with previous work documenting disruptions to the effectiveness of emotion regulation under conditions of sleep loss [21]. During the *decrease/negative* trials of this task, sleep deprived individuals had more difficulty actively regulating emotional responses to negative stimuli when instructed to do so. Although all subjects were able to down-regulate their experience of affectively negative stimuli, those in the TSD condition showed a smaller down-regulation effect compared to rested controls. Based on results from other tasks in this study, it is unlikely that the change in sleep deprived subjects’ emotion regulation was due to changes in bottom-up processing of affective information, as there was no discernable shift in how affective information was attended to, maintained, or categorized in the non-goal directed portion of the emotion regulation task nor in the other tasks in the study. In other words, the current findings suggest that TSD has no significant impact on how affective information is processed, but rather has a specific effect on the goal-directed regulation of emotion.

The emotion regulation results are consistent with an interpretation of TSD-induced impairment of top-down attentional control processes required for emotion regulation. Recent work indicates that TSD impairs cognitive control, particularly when the tasks require shifting attentional strategies [67,88]. Additionally, differences in cognitive control have been shown to be related to the effectiveness of emotion regulation both behaviorally and through neuroimaging [60,81]. Separately, positive affect has been shown to be important for quick adaptation of control between differing goals [72,89]. Although it is possible that TSD directly affected individuals’ ability to flexibly change the context in which an emotion is represented, our covariate analyses indicated that TSD related changes in mood accounted for the impairments seen on the emotion regulation task. When we added time-varying covariates of mood into our model of the *decrease/negative* condition of the emotion regulation task, the crucial interaction between condition and session was no longer significant. These findings suggest that changes in mood while sleep deprived impact the ability to regulate emotion in a goal-directed manner, a finding consistent with previous research reporting that mood, and positive affect in particular, influences cognitive control processes [31,32,72,89].

Yet, we also found that adding a non-affective self-report measure of sleepiness as a covariate, instead of the mood covariates, produced the same pattern of results. This suggests that the effect of sleep deprivation on emotion regulation is not explained by a change in mood specifically, but rather a change related to both affective and non-affective subjective states during sleep deprivation. As such, our results for emotion regulation could be due to a generic effect of sleep deprivation, potentially associated with reduced arousal [18,90]. How exactly this generic factor would reduce the ability to regulate emotional experience remains to be investigated.

There are some potential limitations to our findings that are worthy of discussion. It is possible that subjects were exposed to specific valenced words multiple times during the course of the experiment, which could have resulted in semantic satiation and a loss of both access to the meaning of these words and a disruption of individuals’ ability to recognize their emotional valence [91]. However, semantic satiation is unlikely to have affected our results, as subjects in our experiment were not exposed to the words with the same frequency or within the same timeframe as traditionally seen in semantic satiation experiments. We also used both affective words and affective images to assess different components of affective processing. Although the use of different forms of affective stimuli was deliberate and allowed us to assess affective processing across different stimuli sets, people generally experience greater emotional reactions to images than to words [92]. Still, we failed to find any interaction of condition by stimulus valence in our vigilance, working memory, and affective categorization tasks, and in the attending portion of the emotional regulation task. Thus, while our stimuli may have had unequal affective valence across tasks due to the repetition of specific words or differences in presentation mode, we do not believe these contributed to the lack of interaction effects between condition and valence on any of our tasks.

For the affective PVT, it is possible that a lack of interaction of condition by stimulus valence could have been the result of subjects responding to the initial flash of light rather than fully processing the word stimulus. However, the processing of words is relatively fast and automatic (for a review, see [93]). Emotional words also show evidence of this automatic processing, even for emotional words presented at extremely short (< 200 ms) intervals [94,95]. In line with these expectations, our *post hoc* analysis found that RTs on the affective PVT were highly related to RTs on the standard PVT but slower overall, and that accounting for performance on the standard PVT accounted for much but not all of the variance associated with performance on the affective PVT. These results likely indicate that subjects were encoding the words as intended. As a result, any differential capture of attention by valence should have still been present.

With regard to the emotion regulation task, we do not know the strategy subjects employed to suppress their emotional responses in the *decrease/negative* condition, and also whether or not TSD may have resulted in a change in strategy. Although subjects were trained to use the reappraisal strategy, it is possible that other emotion regulation strategies were used. We did not probe subjects on the strategy used during this condition, so we cannot be certain that subjects were using reappraisal. Probing subjects to uncover what strategy they used and potentially using more objective measures of experienced emotions based on physiological responses would provide more insight into whether sleep deprived subjects are less able to regulate their emotions. Additionally, comparing different emotion regulation strategies that require differing amounts of cognitive control may help shed light on the role cognitive control plays in regulating one’s emotions under TSD [79,80].

The emotion regulation task may have induced demand effects for the *decrease/negative* condition, such that subjects may have felt the need to rate negative images less negatively than they actually experienced. If this were the case, the observed effect would not be due to a deficit in top-down control of emotion but rather due to diminished effort to conform to what the subjects may have believed the research team wanted to see, since subjects would feel less obliged to rate negative images as less negative. We believe such an explanation in terms of social desirability is unlikely, as standard uses of emotion regulation tasks have not found this effect and often found relationships between ratings and neurological responses [79–81]. Additionally, our TSD subjects did not display any systematic differences in their *attend/neutral* and *attend/negative* ratings of negativity during this task. The observed effect of TSD on the *decrease/negative* ratings is more likely due to TSD-induced changes in cognitive control [67,88,90]. We acknowledge that use of a more definitive method for assessing strategy use, and emotion regulation more generally, would strengthen this claim.

By using multiple tasks with the same subjects in a controlled experimental context and laboratory environment to investigate the effects of TSD on affective processes, this study highlights the importance of differentiating between bottom-up flow of affective information and top-down control of affective processing. Our results indicate that changes in processing of affective information observed under TSD do not reflect increased attention toward emotional information nor any generalized biasing of affective information during TSD. In other words, components related to the bottom-up flow of affective information remain largely unchanged during TSD. Critically, in contrast to past work [33,36,42], we found that sleep deprived individuals do not differ significantly from their rested counterparts in attending to, identifying, updating, and maintaining affective information, indicating that bottom-up flow of affective information is not affected by TSD and that the TSD effects we do observe are likely the result of non-specific deficits unrelated to affective processing. However, our results are in agreement with previous observations that individuals under TSD have greater difficulty engaging in controlled emotion regulation processes when there is a goal to do so [21]. This impairment does not appear to be due to any systematic change in how affective information is processed, but is likely due to TSD effects on higher order control processes that are responsible for goal-directed regulation of emotion rather than a specific deficit in affective processing. In conclusion, our evidence indicates that sleep-loss related changes to emotional processing are not likely due to changes in bottom-up components of affective processing.

## Supporting information

S1 File (CSV)

S2 File (CSV)

S3 File (CSV)

S4 File (TXT)

S5 File (CSV)

S6 File (CSV)

S7 File (CSV)

S8 File (CSV)
